# The Several Ages of Medical Man

**Published:** 1969-01

**Authors:** Alan B. Raper


### THE SEVERAL AGES OF MEDICAL MAN

BY

ALAN B. RAPER

Young men are bumptious and selfish, and old men are bad-tempered and obstructive—not all of them, at either age, but enough of them for us to be quite familiar with the types. What happens in between? And how, in a profession like medicine, can we make the best use of the good qualities of each stage of life, and minimise the effects of the bad qualities? Or, if we cannot alter the situation, how can we adapt to it or make allowances?

Serious entry into the practical profession of medicine occurs round about the age of 25, and it is from that age that we must begin to trace a man's changes. (I speak of men only, leaving an analysis of a woman's professional life to someone else). To a large extent, of course, the years before 25 have already decided what sort of a man each will be throughout life, and what will be his aspirations and guiding principles. For we all build up a picture, a hope, a dream, of what part we shall play in life; and many of our crucial decisions are really answers to the question " Will it give me contentment to *watch* myself playing this part?" But by and large those who have chosen medicine or any similar profession will have many aspirations in common, so that while still very diverse, these young men can be thought of as a group fairly well marked off from all their fellows. Moreover, for good or ill, their profession will increasingly mould them into an ever more compact body, and this justifies us in thinking of them as a whole.

Diversity, then, is greatest in the early years, originating in the influence and the ties of home and family. Who takes over the moulding process from family, school, and university? Not, I think, the professor or senior clinician; these are too remote, they are hardly seen as beings of flesh and blood, they have lived their life, they are out of date, they do not understand, they do not seem to care. But the younger man aged 30 to 35 has the required heroic properties. He radiates competence and success, he is the master of modern knowledge, having only recently shown that he can pass the important examinations. Moreover his state is seen as attainable within a measurable time, and to see it is a spur to effort. And effort comes easily in the twenties. On the whole I think that our young graduates are well served by these idols of their youth.

But when effort begins to produce achievement, the vision perceptibly changes. It is found that to clear the hurdles that bar the way to the various branches of medicine is not the same as to " arrive ". Recognition does not follow immediately, and the competition becomes more intense. A man at age 30 to 35 is at the height of his physical endurance, and nearing the time when intellect and practical ability make their greatest contribution to professional knowledge. Feeling that he is competent and knowledgeable, but perhaps not aware that the fruits of experience have yet to be gathered and digested, and concentrating all his attention upon himself, he needs considerable balance to appreciate that others may not accept his own estimate of himself and his future. He is fortunate if, as his powers mature, he does not meet with real or imagined obstruction, or, if he meets these obstacles, he does not feel aggrieved. This is indeed a critical time. What is necessary is that a young man of ability should receive almost daily from his seniors some proof that his ability is appreciated; and the best way of providing this is to allow him responsibility—certainly in clinical matters, and to some extent for the training of his juniors. Only by taking clincial responsibility can he begin to realise that academic knowledge is but the beginning of his professional dealings with men and women.

There is a dilemma here, and it relates to research. In the natural sciences, it may well be that a man of 30 to 35 is already at his best, or even beyond it, in developing new lines of thought. In medicine, considered as science, this must be equally true; but in clinical research the material is human beings, and what may happen in the natural sciences is not a sufficient guide. Understanding and compassion are needed. Young men are not devoid of these, but it seems to me that it is made difficult for them to exercise these virtues if they are encouraged to undertake investigative medicine divorced from responsibility for the patient's welfare or, still worse, to indulge in investigations without a clear plan as to what is expected to be achieved. Too much of this sort of investigation is condoned in hospitals. It is the responsibility of a young man's seniors to control and canalise his energies; to allow him the maximum freedom to form his own opinions, while at the same time employing tact in helping him to modify, and indeed mollify them. A good solution at this period is that a flair for research should be directed into the more basic and less clinical aspects of medicine.

The years from 35 to 45 are the years of greatest production. If a helping hand was needed at the beginning of this period, it soon becomes superfluous as a man assumes his professional independence. Towards the end of the period, indeed, a reversal of trends begins. Self-advancement begins to lose its pride of place. Marriage itself contributes little to this change, but the coming of a family certainly does, for to many men this means an introduction to the idea that one must give as well as take; and this attitude begins to find its way into a man's relations with his colleagues. But it is opposed by male combativeness. Intolerance, haste, and pungacity all persist. They have their value, especially in the furthering of quite laudable ambitions. However, it is reasonable to expect that by the age of 40 a man has made a fairly clear and honest assessment of what he can and cannot do. Before this, time and his capacity for achievement seemed infinite, whereas he should now realise that both are limited, and this knowledge should moderate his personal ambition and enlarge his toleration. Confident in his own strength he is now in a position to see both backwards and forwards, to help his juniors and to see some of the difficulties of his seniors. This adjustment is not made equally by all. It is my view that if it has occurred — and his colleagues and seniors can readily judge whether a man has attained this sort of maturity — now is the time to offer a man positions of leadership and power. The structure of the medical profession in this country does not allow enough in the way of openings for such men but much could be done by those who serve on appointments boards if they would reach out for the youngest and best.

Those who have to choose relatively young men for promotion have a delicate task. They must balance their assessment between a man's achievements and his promise. If they misjudge the latter, they may commit an institution to irrevocable domination by a second-rater. If they rely on the former, their choice may be a timid one, for they are likely to choose a older man whose peak of activity has passed; but of course they may do this purposely, if they are looking for stability and judgement rather than activity. As for the many men who are not chosen for the highest posts, they are bound to suffer disappointment and a jar to their pride, unless they have already decided that it would be wiser not to contend. To a large extent the degree of their disappointment will be a measure of their failure in self-assessment. The world is not perfect and justice is not always done, but resentment and disaffection that stem from disappointment at this age almost always indicate an incompletely adjusted personality. If a man has to suffer this sort of check, he is fortunate if he suffers it early, while he has still some adaptability and can re-shape his course.

The years from 45 to 55 are usually more settled. But life brings new problems. A new world, the world of the young, gradually separates itself, and a man realises, with more or less melancholy, that he does not belong to it. But he has plenty of contacts with it—his own children and perhaps students or young graduates. From these he can learn much. Not that he can enter their world, but he can at least see that its inhabitants are eager and well-intentioned, and if self-willed, disarmingly innocent. His views, which were crystallising, are for the moment re-dissolved, and can mix and react with those of the young. He has now no serious problems in relation to his profession. All that is required of him he can do easily, and he finds (somewhat to his surprise, for he still thinks of himself as young) that quite alarming deference is shown to his opinions both by patients and colleagues. But if amidst his busy life and crowding commitments he stops to think about it, he will realise that some of his facility comes from withdrawing (slightly at first but increasingly) from the things that he cannot do so well, and keeping upon the paths that he has come to know intimately. He begins to delegate authority in some matters. In fact he must, for often new burdens in the public service are thrown upon him. It is proper that he should accept these, for he never was, and never will be again, so able to give guidance and apply himself energetically to turning decisions into realities. Moreover this time of life is the ripest for the despatch of business. A full measure of this burden should be carried now, while it can be enjoyed. But it is important that it should not be retained after zest and ability have begun to slacken.

Some time between 55 and 60, a man begins to admit that he is growing older. Of course, he sees the signs first in his contemporaries, but in time detects them even in himself. The process is gradual, and men vary in the age at which it starts; they also vary in the sensitivity with which they recognize it. In general, it is easier to recognize failing powers in purely professional matters; the assimilation of new work becomes more difficult, and the initiation of new lines of thought becomes more burdensome. Association with younger doctors ensures that the older man is kept in contact with the changing scene in medicine, but in reality this is deceptive, for it is hard to recognize and admit how much one is parasitic upon younger minds and more vigorous bodies. Despite this, judgment remains and can be most helpfully deployed. In matters of policy and administration, however, failing powers are less easily noticed. Moreover the desire for power lingers, and in our present professional structure the opportunities for gratifying it are kept open. The power that we are considering here is of two sorts. On the one hand it affects the day-to-day work of a man's juniors, even—although indirectly—extending its effects into their personal lives; on the other hand it affects the present organisation and the future policy and plans of whatever institution a man works in, whether it be a practice, a laboratory, or a university department. To regulate his affairs with his juniors, a man needs but ordinary humanity and decency, and this is hardly to be judged as a professional affair. But in controlling an administrative machine, his prejudices, his recoil from new ideas, and his general lack of confidence in the future, may all outweigh what he can contribute in experience and wisdom. Of course this does not always happen, and many elderly men have combined their accumulated experience with an enlightened, generous, and active outlook. But it would be preferable to provide an avenue whereby, around the age of 60, a man could begin to relinquish some of his power to control the present and shape the future.

The problem raised above needs careful thought. Matters concerned with money loom large. Very few doctors today can have acquired even a modest fortune in their middle years. The later years of professional life coincide with the years of heaviest expenditure on one's children, and a man feels that he must cling to the highest salary, and earn the highest pension that he can obtain regardless of how efficiently he now works for it. To fix a bar at 65, which is the general rule in Britain, may be administratively convenient, but it is stultifying. A few men could easily carry their full load for another five years. Many could continue to carry a light and familiar part of their load well beyond the age of 65. Most would be glad, and in the interests of their juniors would be well-advised, to shed responsibility earlier, but to retain a restricted interest in their profession and receive a lower salary up to and beyond the present bar.

To cater for these various needs would not be easy, but it is not impossible; and to neglect to do so is to misdirect professional talent. The administrators of a salaried service, national or university, could well address themselves to the problem of utilising earlier the drive of the not-so-old, and prolonging the restricted usefulness of the elderly. They could, at least for medicine, take some of the sting from the epigram " Si jeunesse savait, si vieillesse pouvait ".

